# Grass composition and distribution patterns as determinants of behavioral activities and weight accumulation of Nguni and Boran cattle post-relocation

**DOI:** 10.3389/fvets.2022.926140

**Published:** 2022-11-25

**Authors:** Mhlangabezi Slayi, Leocadia Zhou, Yonela Zifikile Njisane

**Affiliations:** ^1^Risk and Vulnerability Science Centre, University of Fort Hare, Alice, South Africa; ^2^Department of Livestock and Pasture Science, Faculty of Science and Agriculture, University of Fort Hare, Alice, South Africa

**Keywords:** novel environment, beef cattle, climate change, foraging behavior, rangelands

## Abstract

Grass biomass composition and distribution patterns within the paddock as determinants of behavioral activities and animal performance of Nguni (NG) and Boran (BR) cattle post-relocation to a novel environment were examined. Ten steers of each breed aged 9 months were bought from two different farms and sent to Honeydale research facilities, where they were reared on rangelands for 12 weeks. Identification and classification of grass species were done every sampling week before introducing cattle to each paddock. Direct visual observations and durations of behavior and paddock occupancy patterns were recorded every fortnight between 0500 and 1900 h every week. Individual animal weights and body condition scores (BCS) were recorded two times per week. Location within paddocks hugely affected (*P* < 0.0001) the composition of the vegetation as most grass species were found everywhere on pastures, near the watering points and along fencelines. However, the distribution patterns of the grass species significantly differed at different locations. *Aristida congesta* was dominant (P = 0.0014) everywhere in the pasture and along fenceline than in areas with a high density of trees. Except in shaded areas, *Cynodon dactylon* (*P* = 0.0003) and *Eragrostis chloromelas* (*P* = 0.0008) were highly abundant near the watering points, pastures, and along the fenceline. *Themeda triandra* (*P* < 0.0001) was only prevalent everywhere on pastures except in shade areas, near the water sites, and along fenceline. In terms of palatability and ecological groups, highly palatable species (*P* < 0.0001) and decreasers (*P* = 0.0010) were more frequent everywhere in the paddocks. From Weeks 1 to 3, NG spent more time walking (*P* < 0.0001), while the BR showed a significant decline in grazing activities (*P* < 0.0001) in spite of several differences in vegetation composition. Both breeds showed a significant decline in weight gain (*P* < 0.0001) and body condition score (*P* < 0.0001) in the first 3 weeks. However, the two cattle breeds quickly compensated for their behavioral activities and weight gain, and this shows a good ability to cope with stress caused by heterogeneous environmental conditions.

## Introduction

A cattle ranching holds a significant contribution as a tool to alleviate the poverty crisis among the disadvantaged rural people in South Africa and beyond (1), where three-quarters of the population primarily relies on livestock farming as a source of income (2). Seventy-five percent of cattle production in the country is undertaken in areas, where land and the surrounding environments are harsh and not suitable for other viable use (3). Cattle production in rural areas basically relies on natural pastures as a source of feed (4). Herbaceous forages, browse species, and crop residues are the main feed resources offering a range of goods and services, such as cattle grazing, an important component of extensive livestock production (5). In South Africa, normally cattle production has two distinct seasons: rainy and dry seasons (6). During the rainy season, from October to March, cattle typically have a reasonable amount of forage available (7). Conversely, during the dry season, from April to September, pastures often present restricted forage quantity and quality (8). Thus, the nutritional and productivity of natural pastures throughout the year are not enough for grazing animals to reach their full productive potential (9). Under such challenging situations, cattle farmers are forced to establish supplementary plans depending on the production goals and socio-economic status of the farmer (10). Supplementation involves additional costs to the farmer (8). In most instances, only 20%, if not less, of the farmers could afford the additional feed costs and other management inputs due to the consistent lack of a sustainable income source (11).

Animal performance is dependent on forage intake, which, in turn, is influenced by consumption patterns (6). In heterogeneous pastures such as native grassland, animals change the mechanisms of forage harvest and ingestion, keeping nutrient supply constant (12), and similar behavior may occur when animals have access to better quality pasture. In turn, pastures with higher fiber content cause the animal to reduce forage consumption (13). When it is possible for the animal to select a better diet quality, changes occur in patterns of ingestion and rumination of grazing animals (9). For this reason, foraging behavior is one of the prominent activities in extensively reared cattle as it affords them a better opportunity to learn about their environment by searching for better quality forage, locating water sources (13, 14), and engaging with their herd mates to build social relationships (15). For ruminants, grazing and ruminating activities are essential in nutrient capture and, ultimately, animal performance (16). Up to date, little is known about how the fobs and grass biomass composition at different locations within a paddock may impact different behavioral activities and weight accumulation where animals' food base is natural pastures. A better understanding on how grass biomass composition and distribution patterns determine grazing activities and weight accumulation is important for managing both animals, and their rangelands as climate change through recurrent droughts continue to be an area of concern to many countries (17).

At the same time, high-producing cattle breeds like Simmental, Limousine, and others are experiencing difficulties to adapt to harsh environmental conditions with less available feed resources than their area of origin (13). Consequently, livestock ownership changes involving the relocation of cattle became a norm as farmers were forced to sell a portion of the stock to reduce drought-related financial and production losses (15). Tropical cattle breeds like Nguni and Boran are highly reputed for their ability to strive under nutritive restrictive environments where hot and humid conditions impede productivity (18). For this reason, tropical breeds like Nguni and Boran were allowed to enter the growing commercial sector and extensive recording facilitated breed improvement (19). Thus, while the breeds were improved in the commercial sector, they were being eroded in the rural areas (20). Fortunately, the inherent hardiness of the breeds allowed them to survive, and purebred animals are still found in limited numbers in rural communities. In an attempt to address this problem, there has been a growing interest calling for the mainstreaming of indigenous cattle breeds as a climate-resilient model to improve the tolerance and herd productivity in communal farming setups (21). With climate change impacts occurring at a faster rate than predicted, the current study sought to deposit a portion of the information by examining the distribution and vegetation composition as determinants of foraging behavior and weight accumulation of Nguni and Boran cattle post-relocation to a novel environment.

## Materials and methods

### Ethical approval

Accommodation and care of animals were in accordance with the recommendations of the University of Fort Hare's Research Ethics Policy. The project guidelines were reviewed and permitted under the ethical clearance certificate number MUC551SSLA01 from the Institutional Animal Research Ethics Committee.

### Source of animals and description of experimental site

**Figure 1:**
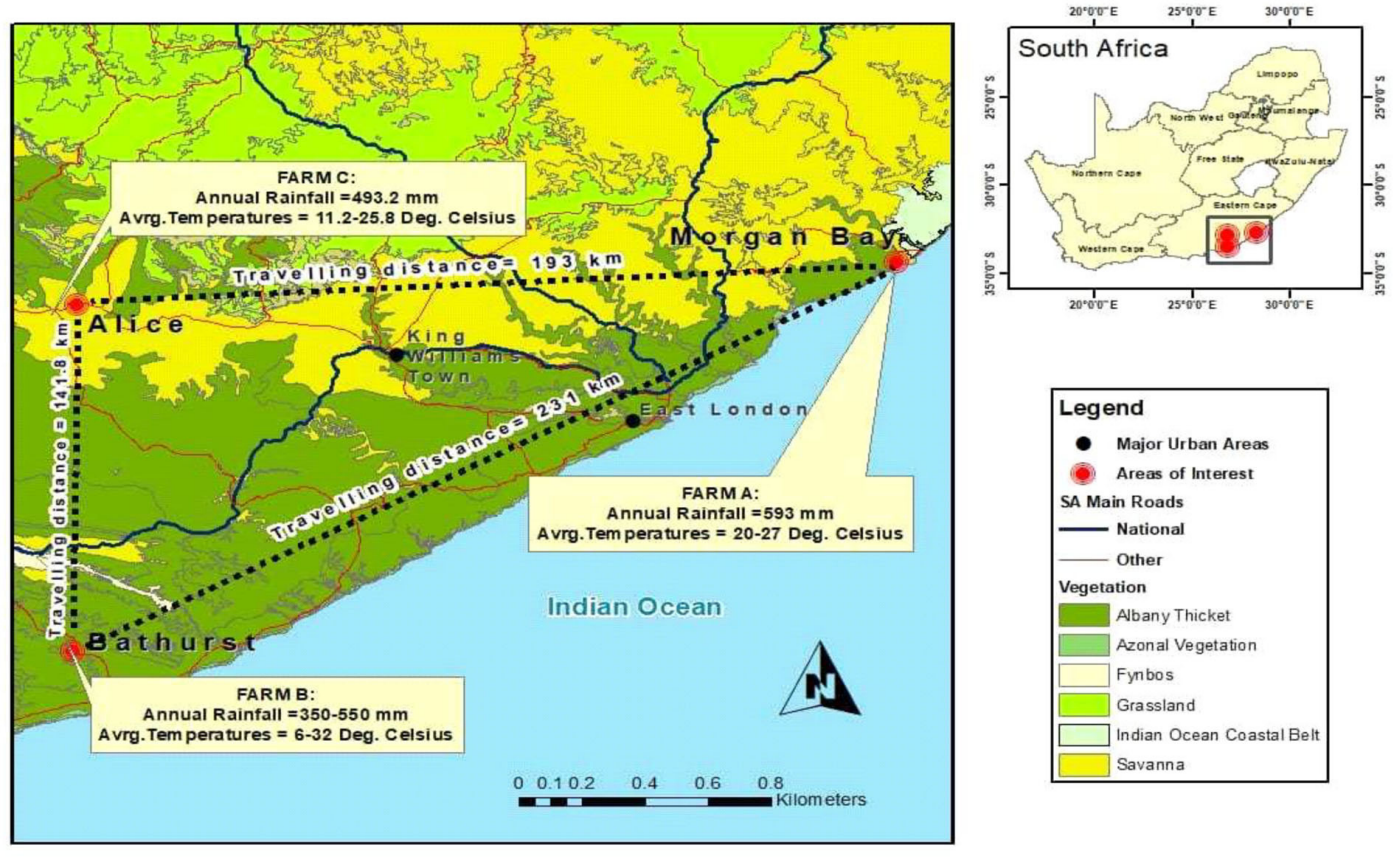
Geographic location of the three farms that participated in the current project.

The animals were sourced from two different farms in the Eastern Cape (Nguni bought in Morgan Bay while the Boran was bought in Bathurst) and sent to Honeydale Research farm at the University of Fort Hare in Alice, South Africa (Figure 1). The Nguni cattle are widely acknowledged to be the outstanding beef breed for optimal production under harsh African conditions. Nguni cattle are heat and light tolerant and can handle extreme heat and cold alike (3). They are adaptable and hardy and possess excellent resistance to internal and external parasites with natural immunity to tick-borne diseases (10). It is slightly smaller in size compared to the large beef breeds of other countries but this just enables it to live in the Highveld regions of Africa (21). Bulls are medium sized and weigh between 500 and 600 kg. They are muscular and display typical male characteristics with well-developed, muscular cervicothoracic humps, which mean that the hump is in front of the foreleg. The cows are small and weigh between 300 and 400 kg. They are feminine with sleek, delicate lines around the neck and forequarter, and a prominent wedge shape with the weight in the stomach and hindquarter area. The sloping rump is a distinctive characteristic of the Nguni cow and ensures ease of calving. The udder is small to medium, well-attached with small, functional teats. On the other hand, the Boran cattle are a medium-sized beef animal. They can be gray, fawn, or red in color. They are recognized for their high fertility, good mothering ability, excellent temperament, and great survivability under harsh conditions (22). Their early maturity and good meat quality will ensure their value in crossbreeding projects aimed at improving the productivity of beef herds. The experimental area is located at 32.8° latitude and 26.9° longitude at approximately 520 m above the sea level. The local climate is classified as semi-arid, with a mean annual temperature of 28.7°C and a mean annual rainfall of 453 mm. The experimental site is 210 ha, which was divided into 36 paddocks of 5.84 ha each. These paddocks were characterized by high heterogeneity in cattle grazing patterns, with some areas showing some signs of degradation and encroachment. The soils in the area are comprised of deep alluvial-derived types in arable lands, which are mostly shallow with <450-mm depth (23). The area lies in the lowland characterized by steep, isolated mountains, and hills with several dams and water streams. The ecological area and veld type predominantly belong to the Bhisho Thornveld (24). The vegetation is composed of several trees, shrubs, and grass species, with *Vachelia karroo, Themeda triandra, Digitaria eriantha, Eragrostis spp*., and *Pennisetum clandestinum* being the dominant plant species (25).

### Experimental design and animal management

Upon arrival at the experimental farm, the steers were supplemented with Vitamin B complex and inoculated with Blanthrax® against Anthrax and other related diseases are known to be prevalent in the area. The steers were allocated into one paddock (500 × 500 m per paddock, length × width) irrespective of their source of origin to allow the acclimatization and buildup of social relationships with their new herd mates. A 6-month resting period was applied to each paddock to ensure there is sufficient forage before introducing the animals. A 24-h accessible watering system was located at the center of each paddock. Different numbered and colored ear tags (green for Boran and white for Nguni) were fitted on each animal for identification. An individual code was marked on the back of each steer using a washable dye to differentiate between steers with the same body color during behavioral observations. The steers were dipped with DRASTIC DEADLINE® fortnightly, as is the new requirement to control tick infestation in the area. The steers had free access to water and were allowed to free-range together throughout the trial period. Rotational grazing was adopted for feeding the animals, and they were moved to a new paddock after every 14 days. A total number of six paddocks were used to carry the animals in the current study.

### Vegetation assessments and species identification

The species composition of the vegetation was estimated from different locations within a paddock using a step-point method (26). The nearest plant and basal strikes were recorded from 250 point observations per location. The point observations were placed at 2-m intervals, and records were made over the length of the plot in five straight parallel lines with a distance of 4 m between them. Grasses were classified based on the succession and ecological information for the arid and semi-arid regions of South Africa (27) as follows: (i) highly palatable species; those that develop on rangeland in good condition and decrease with high grazing pressure (decreaser), (ii) palatable species; those that appear in rangeland in good condition and increase with moderate grazing pressure (increaser IIa), and (iii) less palatable species; those that occur in rangeland in good condition and increase with severe utilization (increaser IIb and IIc). Those grasses that could not be identified were collected with full inflorescences and sent to the South African National Biodiversity Institute (SANBI) Herbarium in Pretoria for identification. In each location, four 0.25 m^2^ quadrants were randomly placed for above-ground grass biomass sampling. Forages within each quadrant were harvested to a stubble height, bulked, and oven dried for 48 h at 60°C. Dried samples were weighed to measure the dry matter (DM) yield.

### Inter-observer reliability and recording of foraging and drinking behavior

Five observers with more than 3 years of participation in data collection involving behavior monitoring were selected in the current experiment. At the beginning of the trial, definitions of behaviors were set, and the observers were trained to gather data and were subjected to a preliminary trial as a protocol to test their understanding and to ensure inter-observer reliability. Using data collection sheets, the five trained personnel recorded the animals' behavioral patterns from 0500 h in the morning to 1,900 h the same day. The procedure was done on three consecutive days each observation week. The same data collectors were used throughout the trial period. Time spent grazing (mouth in contact with grass species), drinking water (head pointing down and mouth in contact with water at the watering point), standing idle or either ruminating, lying down (body in contact with the ground using either lateral or sternal recumbency), browsing (mouth in contact with forage browse tree or shrub) and walking (moving from one place to the next with head raised up) was recorded for every first 20 min of every hour through the scan sampling procedure by Martin and Bateson (28). The location of the animals in the paddock (shade, pasture, fenceline, or water source) was recorded every 20 min per hour. The animal was considered in the shade when the head and most of its body were covered by the shade, at the water source and fenceline, and when standing or lying at <5 m from the water source and fence. Amount of time recorded for each activity and location was expressed as a percentage of the total time spent for all the activities by the animals in the veld. Using the same five trained observers, drinking frequencies were recorded continuously and simultaneously for each animal. The number of instances for each drinking action (drinking bouts) and time spent by each animal drinking water was recorded using a stopwatch. “Drinking bouts” were defined as when at least 4 min was spent without any drinking.

### Live weight and body condition scores

The steers were driven into the handling facility to collect individual animal weights and BCS two times per week. Animals were individually weighed using a digital weighing band (individual scale CAUDURO 40100–1,500 kg, Cachoeira do Sul; Brazil), and live weights were recorded. The steers were weighed at 8 o'clock, after 3 h of fasting. The animals were palpated, and body condition scoring was assessed by experienced personnel using a 1–5 point scale (3). These measurements were taken every fortnight. The average daily gain (ADG) was determined by the difference between weights on Weeks 1, 3, 5, 7, 9, and 12 divided by the number of days between each measurement.

### Statistical analysis

The data obtained were checked for homogeneity of variance and the presence of outliers using the extreme observation table, while normality was tested using PROC UNIVARIATE. Data on foraging activities were arc sin transformed, while the BCS were square-root transformed before the generalized linear model analysis was run. This adjustment was made to ensure the normality of the data. Outliers were set to missing, and the analysis was re-run to determine if any new outliers appeared. This process was repeated until all outliers were removed from the data set. A comparison of means was made using the PDIFF option. The model used was as follows:


$$\begin{array}{l} \text{Yijl}=\text{u}+\alpha \text{i}+\;\beta \text{j}+\text{ hk}+\text{ αβij }+\;\alpha \text{hij }+\;\beta \text{hjl}+\text{ Eijk}, \end{array}$$

where, Y_ijl_ = proportion of time spent on different behavioral activities (grazing, browsing, resting, walking, pasture, shade, fenceline, and watering points); water intake (number and duration of drinking bouts); and animal performance (ADG and BCS. Whereas, μ = overall mean; α_i_ = effect of breed (i = Boran, Nguni); βj = effect of observation week (1, 3, 5, 7, 9, and 12); hk = effect of observer (1, 2, 3, 4, and 5); αβ_ij_ = interaction of genotype and observation week; αh_ik_ = interaction of breed and observer; βh_jk_ = interaction of observation week and observer; E_ijk_ = random errors. Pearson correlation coefficients for the two breeds were used to determine the relationship among the tested variables.

## Results

### Abundance of common grass species, ecological, and palatability groups

The current study identified six common grass species at different locations within the paddocks during the trial period (Table 1). All the common grass species were more abundant except the *Sporobolus fimbriatus*. However, the distribution patterns of the grass species significantly differed at different locations. *Aristida congesta* was dominant (*P* = 0.0014) everywhere in the pasture and along fenceline than in areas with a high density of trees. Except in shaded areas, *Cynodon dactylon* (*P* = 0.0003) and *Eragrostis chloromelas* (*P* = 0.0008) were highly abundant near the watering points, pastures, and along the fenceline. *Themeda triandra* (*P* ≤ 0.0001) was only prevalent everywhere on pastures except in shade areas, near the water sites, and along the fenceline. In terms of palatability and ecological groups, highly palatable species (*P* ≤ 0.0001) and decreasers (*P* = 0.0010) were more frequent everywhere in the paddocks. Biomass production significantly varied (*P* < 0.0001) between location sites, with more DM yield found everywhere on pasture than in other areas.

**Table 1:** Relative abundance (%) of common grass species, ecological, and desirability groups at different locations within a paddock.

**Grass species**	**Water points**	**Pastures**	**Shade**	**Fenceline**	**M.S.E**	**Probability**
*Aristida congesta*	2.94^b^	5.88^a^	1.47^c^	2.94^b^	1.42	0.0014
*Cynodon dactylon*	5.88^b^	7.35^a^	2.94^c^	4.41^c^	1.65	0.0003
*Digitaria eriantha*	2.94^c^	8.82^a^	2.94^c^	4.41^b^	1.44	<0.0001
*Eragrostis chloromelas*	5.88^b^	7.35^a^	1.47^d^	2.94^c^	2.37	0.0008
*Sporobolus fimbriatus*	1.47^b^	2.94^a^	1.47^b^	1.47^b^	1.63	0.0322
*Themeda triandra*	1.47^c^	10.29^a^	2.94^b^	1.47^c^	3.05	<0.0001
**Ecological groups**						
Decreasers	9.38^c^	18.75^a^	7.29^c^	12.50^b^	1.51	0.0010
Increaser I	-	2.08	-	-	0.10	<0.0001
Increaser II	7.29^c^	15.63^a^	6.25^c^	10.42^b^	1.42	0.0002
Increaser III	1.04^c^	3.13^a^	-	2.08^b^	0.71	0.0426
Invaders	1.04^b^	2.08^a^	-	1.04^b^	0.33	0.0500
**Palatability groups**						
Highly palatable	14.14^b^	22.22^a^	8.08^c^	15.15^b^	1.62	<0.0001
Moderately palatable	8.08^b^	19.19^a^	4.04^c^	7.07^b^	1.21	0.0025
Less palatable	3.03^a^	1.01^b^	1.01^b^	1.01^b^	1.24	0.0375
Virtually unpalatable (forbs)	2.02	2.02	-	-	0.81	0.5074

Different letters “a, b, c, and d” represent significant differences (*P* ≤ 0.05) between sampling locations within a paddock.

**Figure 2:**
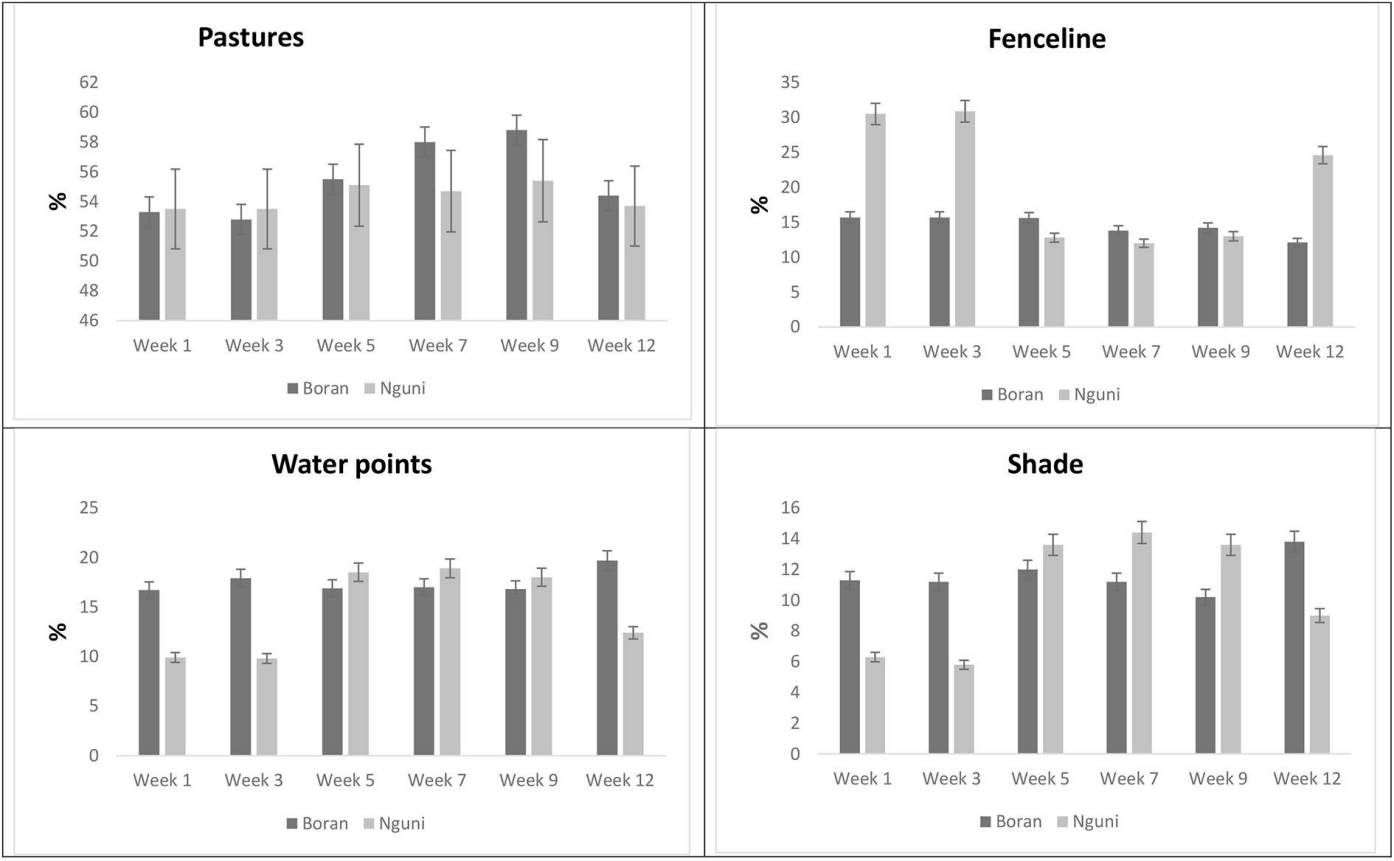
LSMeans for the number and duration of drinking bouts according to breed during the observation period.

Biomass production according to different locations within a paddock is presented in Figure 2. Anywhere within the paddock and fenceline had higher biomass production (*P* < 0.0001) except along the water points and fencelines.

### Behavioral activities displayed by the Nguni and Boran cattle during the observation weeks

The proportion (expressed in percentages) of time spent by the Nguni (NG) and Boran (BR) steers on each behavioral activity during the observation weeks is shown in Table 2. BR had a higher proportion (*P* < 0.0001) of grazing time than NG. However, the proportion of time spent grazing between the two breeds significantly declined (*P* < 0.0001) from Week 1 to Week 5. There was a significant interaction (*P* = 0.0002) between breed and observation period with regard to the proportion time spent on grazing. A higher proportion of time spent on walking events was noted in NG than in BR. The two genotypes showed an increase (*P* < 0.0001) in time spent walking on Week 1 than in the successive weeks. Significant interactions (*P* < 0.0001) existed between breed and observation period with regard to the proportion of time spent walking. BR had higher proportion (*P* < 0.0001) of time spent browsing than NG. Time spent walking by the two breeds showed a significant difference (*P* < 0.0001) with regard to the observation period. A notable decline in browsing activities was observed in Weeks 1–5 than in the subsequent weeks. There was a significant interaction (*P* = 0.0006) between the breed and observation period. The proportion of time spent resting remained insignificantly low (*P* > 0.05) irrespective of the breed and observation period.

**Table 2:** LSMeans for the proportion of time spent on different behavioral activities according to breed and observation period.

		**Period**						**Statistics**			
	**Breed**	**Week 1**	**Week 3**	**Week 5**	**Week 7**	**Week 9**	**Week 12**	**±SEM**	**B**	**P**	**B x P**
Grazing	Boran	44.20^b, x^	39.00^c, y^	42.80^b, y^	50.10^a, x^	51.80^a, y^	51.80^a, x^	1.412	<0.0001	<0.0001	0.0002
	Nguni	35.80^c, y^	40.00^c, y^	44.20^b, y^	42.80^b, y^	49.80^b, y^	43.60^b, y^				
Walking	Boran	33.70^b, x^	38.30^b^	33.60^b^	23.10^c, y^	18.80^d, y^	20.60^c, x^	1.295	<0.0001	<0.0001	<0.0001
	Nguni	42.00^a, y^	38.80^b^	32.60^b^	33.20^b, x^	25.20^c, x^	32.50^b, y^				
Browsing	Boran	12.70^b, x^	12.50^b, x^	13.10^b, x^	18.40^a, x^	16.70^a, x^	18.70^a, x^	0.739	<0.0001	<0.0001	0.0006
	Nguni	11.60^b, x^	11.40^b, x^	13.00^b, x^	13.10^b, y^	14.40^b, y^	13.30^b, y^				
Resting	Boran	9.40^b, y^	10.20^a, x^	10.50^a, x^	8.40^b, y^	12.70^a, x^	8.90^b, y^	0.979	0.4450	0.3499	0.2048
	Nguni	10.60^a, x^	9.80^b, x^	10.20^a, x^	10.90^a, x^	10.60^a, x^	10.60^a, x^				

S.E.M, standard error of means. Different letters “a, b, c” represent significant differences (*P* ≤ 0.05) between periods of observation. “x and y” represent significant differences (*P* ≤ 0.05) between genotypes. B, Breed; P, Period; B x P, Breed x Period interaction.

### Spatial distribution of the Nguni and Boran steers according to observation weeks

**Figure 3:**
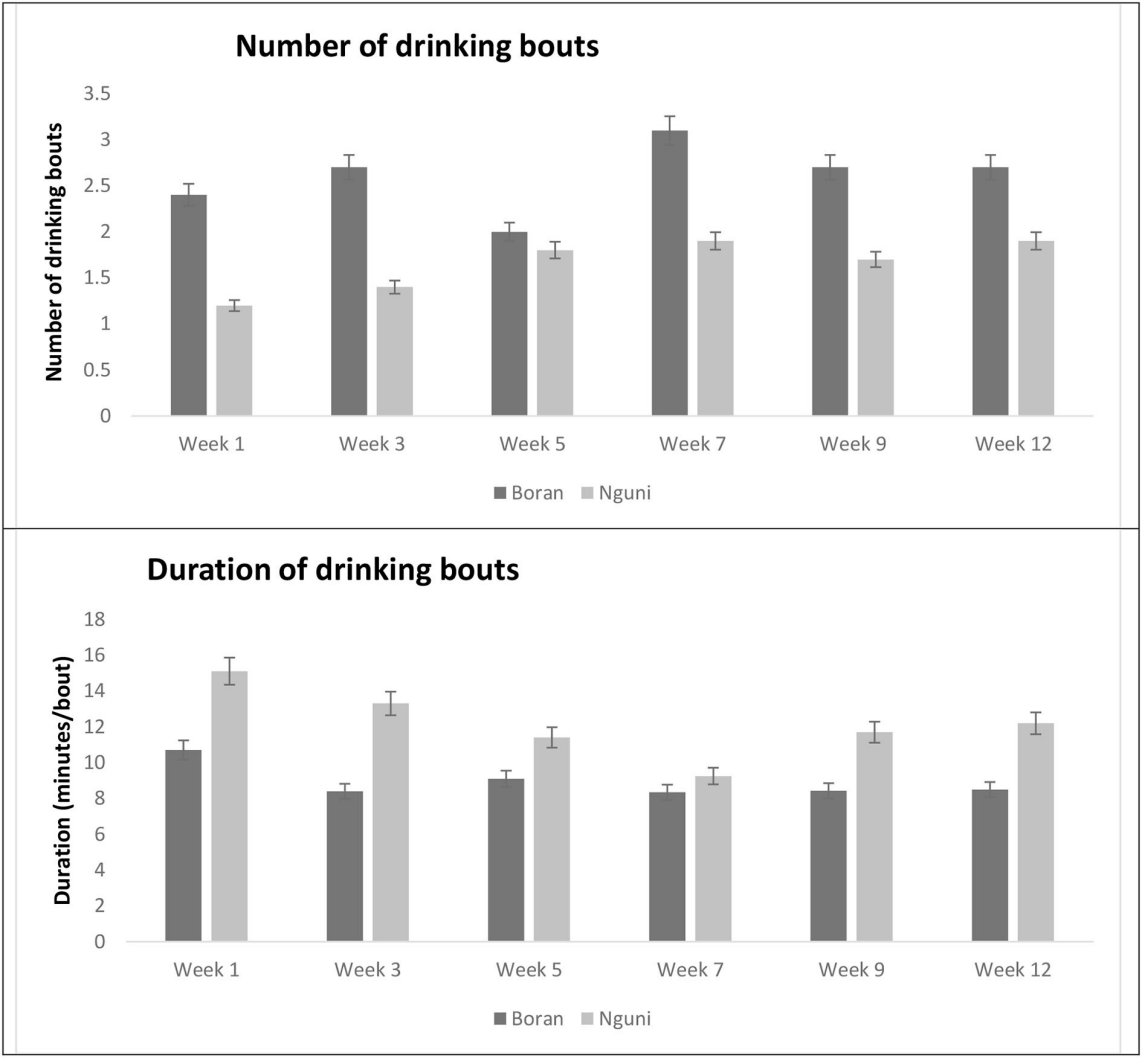
LSMeans for the proportion of time spent on pasture water points, fenceline, and shade according to breed and observation period.

The effect of breed and period on animal distribution in the paddock is presented in Figure 3. The proportion of time spent on pasture remained the same (*P* = 0.0559) irrespective of the breed. However, a significant variation with regard to the time spent on pasture (*P* = 0.0005) was noted within the observation period. From Week 5 to Week 9, the steers spent more time on pasture than in the preceding weeks. NG had a higher (*P* < 0.0001) proportion of time spent along the fenceline than BR. Week 1 and Week 3 showed a higher (*P* < 0.0001) proportion of time spent along fenceline than in the successive weeks. Significant interactions (*P* < 0.0001) were also noted between breed and observation period with regard to the time spent along fenceline. BR spent more (*P* < 0.0001) time along water points than NG. A variation in time spent along water sources was more dominant (*P* < 0.0001) in Weeks 1–3 than in the successive weeks. Genotype and observation period showed a significant interaction (*P* < 0.0001) in response to time spent along water points. The two breeds spent similar time in shade (*P* = 0.1014) during the observation periods. However, the observation period showed a significant variation (*P* = 0.0004) with regard to time spent by the steers near or under the shade. The proportion of time spent under the shade showed significant interaction (*P* < 0.0001) between breed and observation period.

### Water consumption patterns of the Nguni and Boran steers during the observation weeks

**Figure 4:**
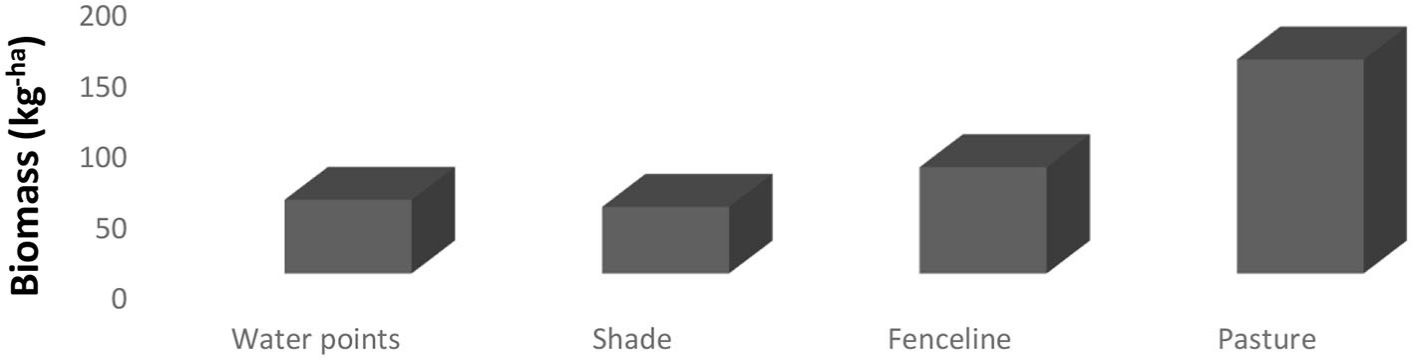
Mean forage biomass production (kg ^ha − 1^) in different locations within a paddock.

No significant interactions were noted between breed and observation period on the number of drinking bouts (*P* = 0.4007) and duration of drinking bouts (*P* = 0.1042) as shown in Figure 4. BR had a higher number (*P* < 0.0001) of drinking bouts than NG throughout the study. Contrastingly, NG had a longer duration (*P* < 0.0001) per drinking bout than BR during the period of behavior observation.

### Average daily weight gain and body condition scores of Nguni and Boran cattle

The average daily gain and BCS of the Nguni and Boran steers are presented in Table 3. BR had higher ADG (*P* < 0.0001) and BCS (*P* < 0.0001) than NG, even though they were of similar age. Both breeds showed a significant decline in ADG (*P* < 0.0001) on Weeks 1 and 3 than Weeks 5–12. On the other hand, the two breeds showed a significant decline (*P* = 0.0028) on BCS during Week 1 only. No significant interactions (*P* = 0.3129) were noted with regard to ADG.

**Table 3:** LSMeans for the average daily gain (ADG) and body condition scores (BCS) according to breed and observation period.

		**Period**						**Statistics**			
	**Breed**	**Week 1**	**Week 3**	**Week 5**	**Week 7**	**Week 9**	**Week 12**	**±SEM**	**B**	**P**	**B × P**
ADG* (kg/day)	Boran	0.36^c, x^	0.38^c.x^	0.47^a.x^	0.50^a, x^	0.46^a, x^	0.43^a, x^	0.012	<0.0001	<0.0001	0.3129
	Nguni	0.30^d, y^	0.31^b, y^	0.41^b, y^	0.41^b, y^	0.42^b, y^	0.35^b, y^				
BCS	Boran	3.00^c, x^	4.00^a, x^	3.70^b, x^	3.70^b, x^	3.60^b, x^	3.50^b, x^	0.181	<0.0001	0.0028	0.1564
	Nguni	2.40^e, y^	2.60^d, y^	2.70^d, y^	3.00^c, y^	3.10^c, y^	2.60^c, y^				

*ADG, average daily gain; BCS, body condition score; s.e.m, standard error of means. Different letters “a, b, c, d, and e” represent significant differences (*P* ≤ 0.05) between periods of observation. “x and y” represent significant differences (*P* ≤ 0.05) between breeds. B, Breed; P, Period; B x P, Breed x Period interaction.

### Correlation coefficients of Nguni and Boran steers among the tested variables

Table 4 shows relationships between all the tested variables for the Boran (top) and Nguni (bottom) diagonal. For BR steers, the proportion of time spent on grazing positively correlated with browsing (*r* = 0.017 at *P* < 0.0001). On the other hand, time spent on grazing activities negatively correlated with resting (*r* = −0.963 at *P* = 0.0025). Browsing negatively correlated with the proportion of time spent on resting (*r* = −0.767 at *P* = 0.0098) and pastures (*r* = −0.699 at *P* = 0.0246). Positive correlations (*r* = 0.634 at *P* = 0.0492) were noted between the proportion of time spent on browsing and fenceline. Resting positively correlated with walking (r = 0.836 at *P* = 0.0026), while negatively correlated (*r* = −0.661 at *P* = 0.0379) with ADG. Walking positively correlated with time spent along the fenceline (*r* = 0.741 at *P* = 0.0142), while negatively correlated (r = −0.701 at *P* = 0.0240) with ADG. The proportion of time spent under the shade negatively correlated with time spent on pasture (*r* = −0.733 at *P* = 0.0158) and fenceline (*r* = −0.780 at *P* = 0.0078). Time spent under the shade positively correlated with time spent around water points (*r* = −0.947 at *P* < 0.0001) and the number of drinking bouts (*r* = 0.782 at *P* = 0.0264).

**Table 4:** Correlation values among the tested variables of interest for the Boran (top) and Nguni (bottom) diagonal.

	**Grazing**	**Browsing**	**Resting**	**Walking**	**Shade**	**Pasture**	**WP**	**FL**	**DB**	**ADG**
Grazing	–	0.017^***^	−0.963^***^	−0.838^**^	−0.146	0.296	0.202	−0.463	−0.289	0.451
Browsing	−0.666^*^	–	−0.767^**^	−0.122	0.127	−0.699^*^	−0.031	0.634^*^	−0.288	0.370
Resting	−0.659^*^	0.479	–	0.836^*^	0.372	−0.183	0.048	−0.241	−0.102	−0.661^*^
Walking	−0.707^*^	−0.750^*^	−0.749^*^	–	−0.291	−0.043	−0.295	0.741^*^	0.588	−0.701^*^
Shade	0.104	0.247	0.439	−0.696^*^	–	−0.733^*^	0.947^***^	−0.780^**^	0.782^**^	−0.250
Pasture	0.191	−0.554	−0.297	0.423	−0.637^**^	–	−0.422	0.931^***^	0.778^**^	0.419
WP	−0.606	0.551	0.161	0.217	0.974^***^	0.080	–	−0.679*	0.701*	−0.794^**^
FL	0.042	−0.147	−0.388	0.396	−0.652^*^	0.684^*^	−0.980^***^	–	0.683^*^	0.219
DB	−0.711^*^	0.396	0.045	0.563	−0.848^**^	0.144	0.701^*^	0.895^***^	–	−0.010
ADG	0.260	−0.696^*^	−0.704^*^	−0.701	−0.308	0.650^*^	−0.173	0.195	0.195	–

Significance at

* (*P* < 0.05),

** (*P* < 0.01),

*** (*P* < 0.001), WP, water point; FL, fenceline; DB, drinking bouts; ADG, average daily gain and BCS, body condition score.

The amount of time spent on pasture positively correlated with time spent along the fenceline (*r* = 0.931 at *P* < 0.0001) and the number of drinking bouts (*r* = 0.778 at *P* = 0.0081). Time spent near the water points negatively correlated with the amount of time spent along the fenceline (*r* = −0.679 at *P* = 0.0308) and ADG (*r* = −0.794 at *P* = 0.0061). Positive correlation (*r* = 0.701 at *P* = 0.0239) existed between the amount of time spent around water points and drinking bouts. The proportion of time spent along the fenceline positively (*r* = 0.683 at *P* = 0.0295) correlated with the number of drinking bouts. For NG steers, the proportion of time spent on grazing negatively correlated with time spent on browsing (*r* = −0.666 at *P* = 0.0357), resting (*r* = −0.659 at *P* = 0.0384), walking (*r* = −0.826 at *P* = 0.0033), number of drinking bouts (*r* = −0.711 at *P* = 0.0210), and BCS (*r* = −0.739 at *P* = 0.0145). Browsing negatively correlated with the amount of time spent on walking (*r* = −0.750 at *P* = 0.0168) and ADG (*r* = −0.696 at *P* = 0.0255). Resting positively correlated with fecal DM (*r* = 0.746 at *P* = 0.0133), while negatively correlated with time spent on walking (*r* = −0.749 at *P* = 0.0125). Positive correlations (*r* = 0.813 at *P* = 0.0042) were noted between the amount of time spent on pastures and a number of drinking bouts. Negative correlations (*r* = −0.696 at *P* = 0.0253) existed between the amount of time spent on walking and under the shade. Time spent under the shade negatively correlated with the amount of time spent on pasture (*r* = −0.637 at *P* = 0.0015), fenceline (*r* = −0.652 at *P* = 0.0411), and drinking bouts (*r* = −0.848 at *P* = 0.0020). The proportion of time spent under the shade positively correlated with the amount of time spent along water points (*r* = 974 at *P* < 0.0001) and BCS (*r* = 0.661 at *P* = 0.0376). A positive correlation existed between the amount of time spent on pasture and fenceline. The amount of time along water points positively correlated with the BCS (*r* = 0.646 at *P* = 0.0437), while negatively correlated with time spent along the fenceline. Time spent along the fenceline positively correlated with the number of drinking bouts (*r* = 0.895 at 0.0005), while at the same time negatively correlated (*r* = −0.739 at *P* = 0.0417) with the BCS. ADG positively correlated with the amount of time spent on pasture (*r* = 0.650 at *P* = 0.0419).

## Discussion

Transferring cattle to a new environment results in a heterogeneous use in different parts of the paddock. However, this might be influenced by the vegetation composition and its distribution patterns since most of the animals rely on natural pastures as a source of nutrition. Findings obtained from this study noted differences in distribution patterns of common grass species, with most of them found along water points and everywhere on pastures. Differences in the abundance of grass species within a specified area is highly influenced by a number of factors, such as weather or climate and management practices (17, 29). Such factors are known to influence the grazing patterns and weight accumulation of cattle that solely depend on native grasslands as a source of feed (13). However, behavioral changes and weight accumulation differences between the two cattle breeds could be attributed to a combination of the new environment and vegetation composition. Exposing cattle to a new environment result in temporal disruptions in their foraging routine, water footprint, and weight accumulation as a response mechanism to the imposed stimuli (10). The response of the animals and time needed to adapt differs with each animal species, sex, breed, age, physiological status, and the production system (15, 30). It is well-accepted and documented in the literature that taking animals to a new environment compel them to make substantial changes in their time budgets, and this negatively impacts their welfare and productivity (31, 32). Previous studies done in cattle production used activity time budgets to measure the response as well as to get some insights into the adaptation potential of different breeds to several climate change shocks (10). Nonetheless, there is limited information about how different cattle genotypes learn to adapt when sent to a novel environment, as this has become a norm in the era of climate change. In the current study, the proportion of time spent on walking became the dominant activity shown by the Nguni and Boran steers during the first 3 weeks post-relocation. The difference in time spent on walking could be attributed to the fact that the Nguni cattle are known for their ability to walk long distances in search of grazing sites and water points (18). At the same time, the Nguni cattle are selective grazers and browsers; hence, the need to walk a longer distance to search for better quality forage, while the Boran are effective grazers (21). Some studies argue that an increase in locomotion may reflect social instability and curiosity of animals to cope with environmental changes (33). With this information, a combination of the season through insufficient forage and novelty of the environment might have played a crucial role in elevated time spent walking with intents to search for better quality food as the study was conducted during dry months. Other studies documented the restriction on time spent grazing as another response made by animals in adjusting to their unknown environment (34). Similar results were achieved in the current study, where the amount of time spent on grazing and browsing significantly dropped in the first 3 weeks of introduction to the new area. Breed differences in time spent grazing and browsing could be attributed to different feeding mechanisms shown by the steers. The remarkable ability shown by the Nguni and Boran cattle to obtain the nutritional value from the available natural vegetation proves to be very beneficial to their excellent adaptation under challenging conditions which could be counterproductive to bulk grazers such as exotic cattle breeds (12).

The proportion of time spent resting remains unchanged in both breeds throughout the study, which could be due to the fact that Nguni and Boran cattle are energetic cattle breeds capable to of adapting harsh climatic conditions and extreme temperatures (3). The two cattle breeds spent equal time on pastures, and this could be due to the fact that cattle are social animals and prefer to graze in groups. Without the social support of familiar group members, individuals may feel vulnerable to predation and spend more time moving up and down as they do not feel safe to rest (20). Similar findings were reported by Barbieri et al. (35), who noted that during the day, cattle spent most of their time grazing in groups. However, the time spent by the steers on pasture appears to be determined by various factors like weather conditions, social relationships of the animals, and forage availability (36). Introducing cattle to an unfamiliar environment results in an unstable social and foraging routine and cattle display a high proportion of heterogeneous patterns with regard to the occupancy distribution (5). For instance, in the first 3 weeks, the steers spent longer time around fenceline, with Nguni steers showing high proportion than the Boran steers. This implies that individual animals differ in their levels of inquisitiveness and motivation to explore novel situations, which is essential for learning and familiarization with a new environment. Grazing around the fenceline could also be a territorial marking and learning mechanism used by animals to familiarize themselves with their new environment (30). On the other hand, other studies argue that time spent along the fenceline could not be the sole indicator of adaption as it can be influenced by a number of factors, including forage availability within the paddock (14). Since the time spent along the fenceline was only dominant during the first 3 weeks, it is agreed that the steers were in the process of familiarizing themselves with their new environment. However, future studies should bring up the issue of seasonality in relation to forage availability and how this influences the spatial distribution of cattle after being exposed to a novel environment. Moreover, the social structure of the herd during grazing movements should be put into consideration as it also had an influence on the spatial distribution of the steers during the period of behavior observations.

There is a general lack of adapted genetic material suited to the prevailing harsh climatic conditions. For instance, exotic breeds tend to lack the adaptive traits necessary for survival and production in the rigorous environment accompanied by extreme temperatures and low forage availability than their area of origin (18). Even though it was minimal, the steers appeared to share equal chances of seeking shade irrespective of the breed. Tropical cattle breeds like Nguni and Boran are heat tolerant, and their medium body frame appears to be very instrumental to harsh and heterogeneous conditions (1). The availability of shade through trees, in this case, is essential for cattle reared on natural pastures as their absence can reduce animal well-being and subsequently alter their daily routine (37). Other studies claim that cattle prefer to graze or hide in dense areas as a protective mechanism to escape from predators and other mechanical intruders (38). Hiding under the trees or dense areas is an adaptation phenomenon used by cattle to protect and defend themselves against potential threats that are perceived as predation risks (6). Under such conditions, time spent by cattle under the shade primarily depends on the degree of disturbance stimuli (15). Predation risks play a prominent role in shaping the activity patterns of many foraging animals; hence, they resort to shifting some activities over others to avoid or reduce risks or extent of interference in competition for resources (7). In the absence of insufficient shade, cattle spend more of their time around water points (39). Similar observations were made in the current study, where Boran cattle were observed to spend more time around water points than the Nguni steers during different observation weeks. Frequent access and distance of cattle from the water sources depend on daily weather conditions like extreme temperatures, humidity, and wind speed (3, 19). Temperature and humidity have a direct relationship with cattle water consumption (4). Cattle tend lose more water from their bodies through consistent perspiration and defecation resulting from increasing physiological demand due to dehydration hence the need for regular access to water sources. Rearing cattle under natural pastures is accompanied by lots of water availability constraints due to erratic rainfalls, as many cattle farmers rely on natural resources like dams, streams, and ponds (2). As a result, water stress is an area of concern nowadays as most cattle breeds, particularly high-producing or precious genotypes, fail to adapt when sent to low-rainfall areas.

Boran cattle had a higher number of drinking bouts than the Nguni, even though no significant interactions were noted between the breed type and the period of behavior observation. A similar observation was made by Simelane et al. (10) who noted that drinking frequency in cattle could be affected by several factors, including weather conditions. In a study conducted by Williams et al. (39), an elevated degree of THI (temperature-humidity index) resulted in cows drinking more water, spending more time at the drinker, making more visits to the drinker, and competing more at the drinker. A similar observation was made in the current study, where cattle aggressively interacted with each other around the water points. It was noted that the Nguni had longer drinking bouts than the Boran steers. These results are unlikely to be explained by the weather conditions alone but might be related to physiological demand and unstable social relationships between the two cattle breeds. The two cattle breeds showed a significant decline in ADG and BCS even though it was just for the first 3 weeks. The difference in ADG and BCS between the two breeds could be that Boran had higher grazing time while the Nguni had walked longer hours during the first 3 weeks. A similar observation was made by Kabasingiza et al. (40) who recorded an increase in body weight loss in the 1st week's post-relocation. Both breeds quickly compensated for their weight loss in the successive weeks. Scientific evidence showed that the Nguni and Boran cattle performed well-under optimal conditions while the exotics performed poorly under the prevailing management practices of the communal system. Small framed cattle breeds like Nguni and Boran have a lower maintenance requirement which is more easily met by the available forage even during dry months (21). This may be due to the maintenance of a high blood urea when the nitrogen content of the pasture drops. As seen in previous studies by Katiyatiya et al. (3) and Simelane et al. (10), the Nguni maintained a level of 13% in winter while the blood urea levels of the Simmental fell to 7 mgs%, approaching the minimum for proper N balance. However, the authors note that the ability to maintain body condition may be due to adaptation to one or more stress factors.

Negative correlations between time spent grazing and the proportion of time spent on resting and walking. At any given time, the grazing will always be the preferred activity shown by the Boran instead of browsed (3, 19). Nguni steers showed negative correlations between the amount of spent grazing and other variables such as browsing, resting, walking, and a number of drinking bouts. It is widely accepted that under optimal conditions, browsing will always be the first preference activity shown by the Nguni cattle (10, 18). A negative correlation between browsing and ADG could be due to the high proportion of time spent by the Nguni while walking in search of browsing. Depending on the area and nature of vegetation, cattle breeds like Nguni and other browsers like goats tend to walk longer distances in search of browse species, and this is accompanied by lots of energy expenditure hence the weight loss (31, 34).

## Conclusion

The study demonstrated adaptation differences with regard to behavioral activities and occupancy patterns of the Nguni and Boran steers post-relocation could be influenced by a combination of the unfamiliar environment and vegetation composition. This had a negative effect on the weight gain and BCS as the two breeds showed a consistent decline in grazing activities and spent more time walking in the first 3 weeks of exposure. The ability of the Nguni and Boran cattle to respond differently to the stimuli and quickly compensate for the weights implies that the two breeds coped very well in spite of several constraints. Reintroducing the indigenous cattle breeds should be a welcomed idea or possible mitigation approach to improve the tolerance of struggling cattle breeds subjected to harsh and heterogeneous environmental conditions. Nguni and Boran cattle should be prioritized in livestock development and breeding programs to better the genetic capacity of the struggling cattle breeds.

## Data availability statement

The raw data supporting the conclusions of this article will be made available by the authors, without undue reservation.

## Ethics statement

The animal study was reviewed and approved by accommodation and care of animals was in accordance with the recommendations of the University of Fort Hare's Research Ethics Policy. The project guidelines were reviewed and permitted under the ethical clearance certificate number MUC551SSLA01 from the institutional Animal Research Ethics Committee. Written informed consent was obtained from the owners for the participation of their animals in this study.

## Author contributions

Conceptualization: MS and YN. Methodology: LZ and MS. Data curation and writing—original draft preparation: MS. Writing—review and editing: MS, YN, and LZ. All authors have read and agreed to the published version of the manuscript.

## Funding

The financial support received from the National Research Foundation (NRF): Scarce Skills Doctoral Scholarship (Grant UID: 102487), Scarce Skills Postdoctoral Fellowship (Reference: SFP150813137239, UID: 99687), and Project TS64 (UID: 99787) are acknowledged.

## Conflict of interest

The authors declare that the research was conducted in the absence of any commercial or financial relationships that could be construed as a potential conflict of interest.

## Publisher's note

All claims expressed in this article are solely those of the authors and do not necessarily represent those of their affiliated organizations, or those of the publisher, the editors and the reviewers. Any product that may be evaluated in this article, or claim that may be made by its manufacturer, is not guaranteed or endorsed by the publisher.
